# Poisons

**Published:** 1878-11

**Authors:** 


### POISONS.

We all have a great horror of being poisoned, without exactly understanding what it is.

*Poison is a disorganization of flesh, or blood, or both.*

Poisons are of two kinds. One, the result of medicinal agents taken into the stomach or circulation, the other the result of bites or stings of living creatures.

I will now state two ideas, which if generally known, and remembered, would save thousands of lives every year.

If you have swallowed a poison, whether laudanum, arsenic, or other thing poisonous, put a table-spoon of ground mustard in a glass of water, cold or warm, stir and swallow quickly, and instantaneously the contents of the stomach will be thrown up, not allowing the poisonous substance time to be absorbed and taken into the blood, and as soon as vomiting ceases, swallow the white of one or two new eggs, for the purpose of antagonizing any small portion of the poison which may have been left behind. Let the reader remember the principle, which is to get the poison out of you as soon as possible; there are other things which will produce a speedy emetic effect, but the advantage of mustard is, it is always at hand, it acts instantaneously, without any after medicinal effects.

The use of the white of an egg is, that although it does not nullify all poisons, it antagonizes a larger number than any other agent so readily attainable.

But while taking the mustard, or egg, send for a physician; these are advised in order to save time, as the difference of twenty minutes is often death.

